# Supplementary material for: Reducing functionally defective old HSCs alleviates aging-related phenotypes in old recipient mice
Source: Cell Res. 2025 Jan 2;35(1):45–58. doi: 10.1038/s41422-024-01057-5 (PMC11701126; doi:10.1038/s41422-024-01057-5)
Supplement: Supplementary file 1 — Supplementary Figure 1 [file 41422_2024_1057_MOESM1_ESM.pdf]

Supplementary information, Fig. S1

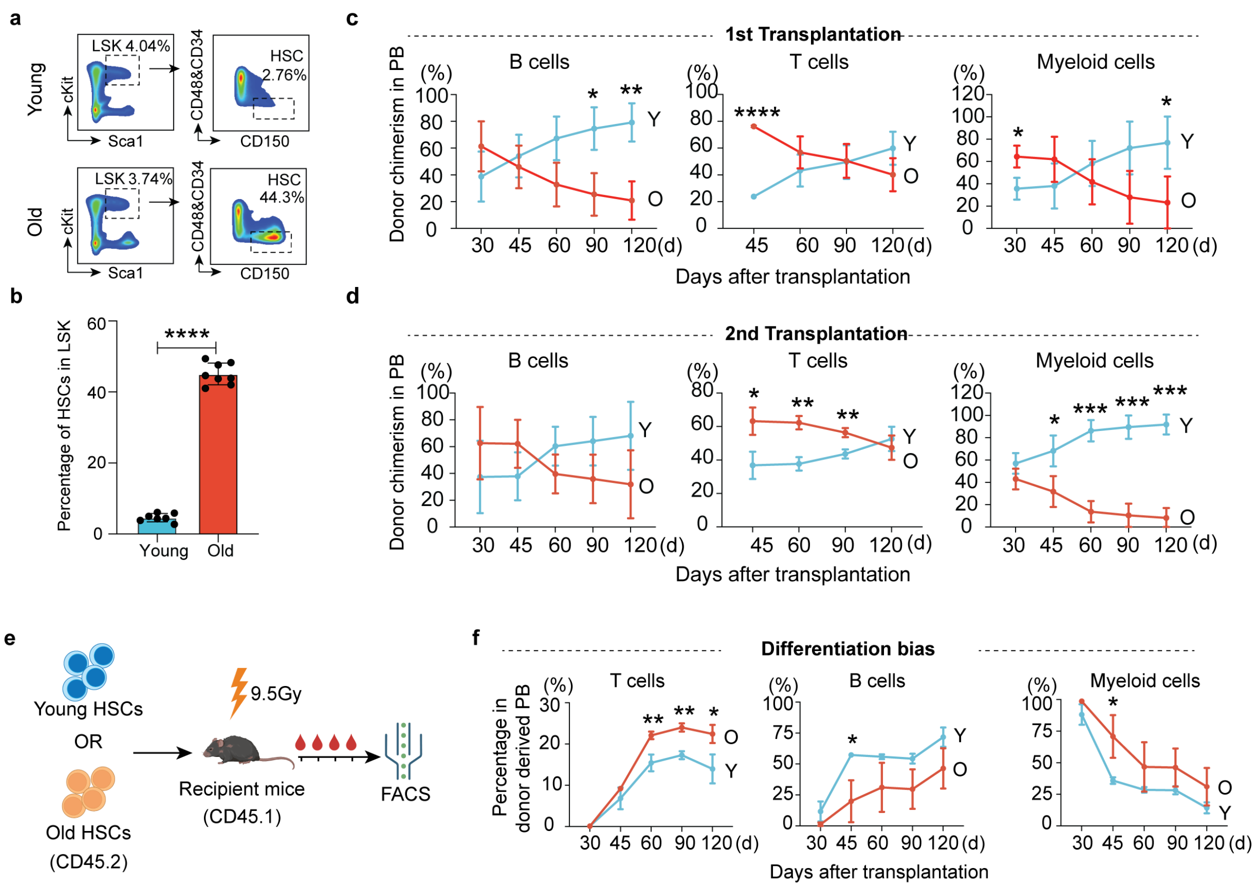

Fig. S1 Aging-related functional decline of HSCs in mouse (related to Fig. 1). a The gating strategy for LT-HSCs sorting. The lineage$^-$Sca-1$^+$c-Kit$^+$(LSK) was first gated, and LT-HSCs (CD48$^-$CD34$^-$CD150$^+$) were gated in LSK population. **b** Bar graph showing the percentage of LT-HSCs in LSK HSPCs from young (2-3 months) and old (22-24 months) mice, *n* = 10 for young, *n* = 9 for old. **c-d** Peripheral blood chimerism of donor HSCs at different time after the first (**c**) and second (**d**) transplantation, *n* = 6 for the first and *n* = 3 for the second transplantation. **e** Diagram illustration of individual transplantation of young and old HSCs into young recipient mice for assessing differentiation bias. **f** Analysis of HSCs differentiation toward T, B and myeloid cells at different time after transplantation, *n* = 3. Mean ± SD, student t test, *P<0.05, ** P<0.01, *** P<0.001, **** P<0.0001. The graphic of the mouse in **e** was created with BioRender.
