# Supplementary material for: Reducing functionally defective old HSCs alleviates aging-related phenotypes in old recipient mice
Source: Cell Res. 2025 Jan 2;35(1):45–58. doi: 10.1038/s41422-024-01057-5 (PMC11701126; doi:10.1038/s41422-024-01057-5)
Supplement: Supplementary file 2 — Supplementary Figure 2 [file 41422_2024_1057_MOESM2_ESM.pdf]

Supplementary information, Fig. S2

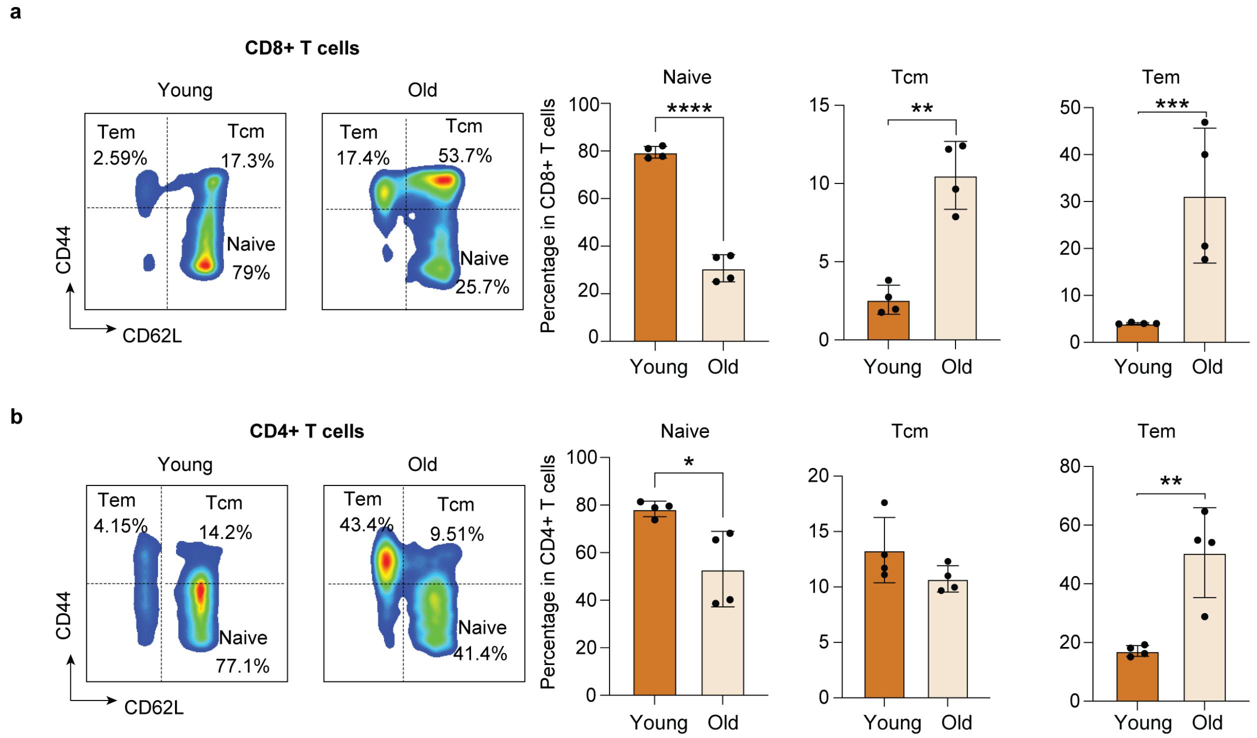

Fig. S2 Immune profile alterations during aging in mice (related to Fig. 1).

a-b Representative FACS analysis and bar plot showing the change in percentage of naïve, Tcm and Tem with aging in PB of mouse in CD8+ T cells (**a**) and CD4+ T cells (**b**). Mean ± SD, student t test, n = 4, * P<0.05, ** P<0.01, *** P<0.001, **** P<0.0001.
