# Supplementary material for: Reducing functionally defective old HSCs alleviates aging-related phenotypes in old recipient mice
Source: Cell Res. 2025 Jan 2;35(1):45–58. doi: 10.1038/s41422-024-01057-5 (PMC11701126; doi:10.1038/s41422-024-01057-5)
Supplement: Supplementary file 3 — Supplementary Figure 3 [file 41422_2024_1057_MOESM3_ESM.pdf]

Supplementary information, Fig. S3

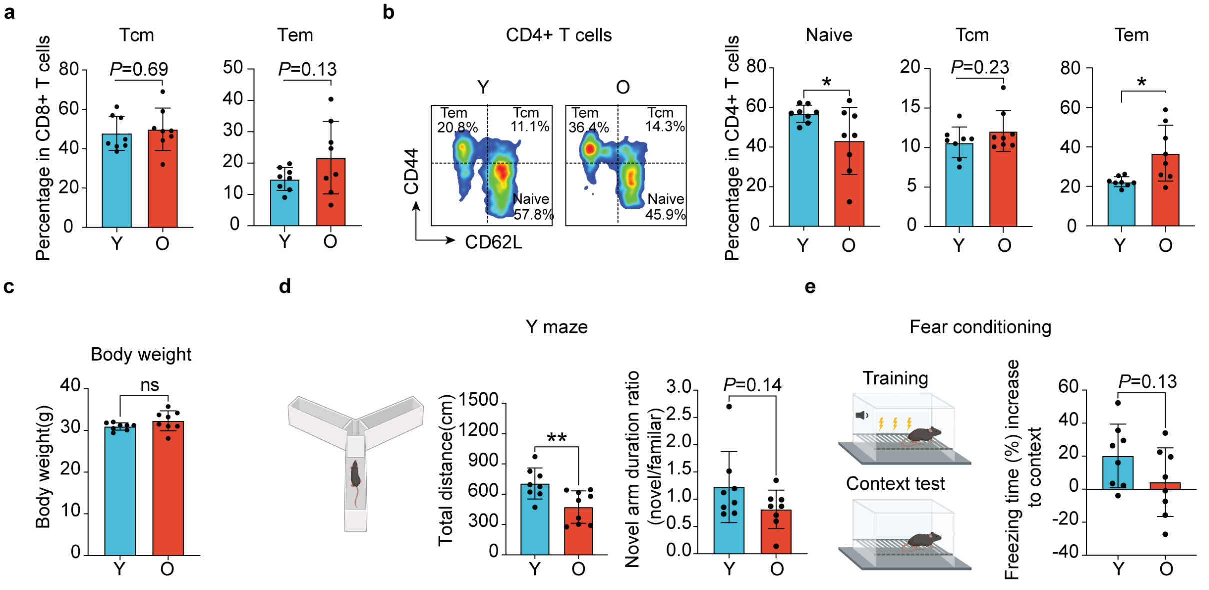

Fig. S3 Transplantation of young HSCs alleviates aging phenotypes in old recipient mice (related to Fig. 1). a Bar plot showing the percentage of Tcm and Tem in CD8+ T cells from recipient mice, *n* = 8. **b** Representative FACS analysis and bar plot showing the percentage of naïve T cells, as well as Tcm and Tem in CD4+ T cells from recipient mice, *n* = 8. **c** Bar plot showing the body weight of recipient mice that received young or old HSCs, *n* = 8. **d** Bar plot showing the total moving distance and the ratio of duration in novel arm to familiar arm in Y maze test of recipient mice, *n* = 8. **e** Bar plot showing the results of context fear conditioning test. Compared to acclimation, the increased freezing time was calculated, *n* = 8. Mean ± SD, student t test, ** *P*<0.01, ns, not significant. The graphic of the mouse and equipment in **d** and **e** were created with BioRender.
