# Supplementary material for: Reducing functionally defective old HSCs alleviates aging-related phenotypes in old recipient mice
Source: Cell Res. 2025 Jan 2;35(1):45–58. doi: 10.1038/s41422-024-01057-5 (PMC11701126; doi:10.1038/s41422-024-01057-5)
Supplement: Supplementary file 4 — Supplementary Figure 4 [file 41422_2024_1057_MOESM4_ESM.pdf]

Supplementary information, Fig. S4

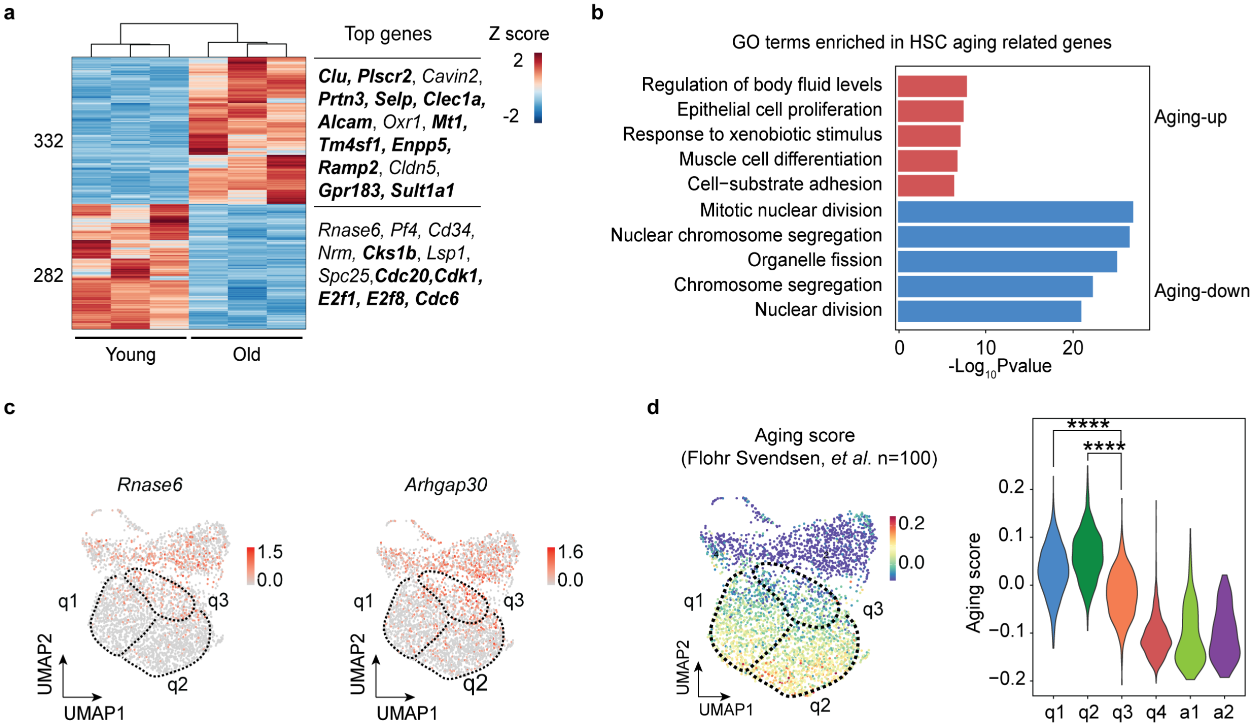

Fig. S4 scRNA-seq reveals increased heterogeneity of old HSCs (related to Fig. 2). a Heatmap showing HSC aging related genes that were identified through bulk RNA-seq analysis. Well-known HSC marker genes in public datasets were highlighted. **b** Bar graph showing the enriched GO terms in HSC aging related up and down genes identified in (a). **c** UMAP showing the expression of the young HSC marker genes (*Rnase6* and *Arhgap30*) in single cells. **d** UMAP (left) showing the calculated aging score based on public HSC aging marker genes (Supplementary information, Table S3) in each single cell. Violin plot (right) showing the aging score of cells from different clusters. Two-sided unpaired Wilcoxon test, **** *P*<0.0001.
