# Supplementary material for: Reducing functionally defective old HSCs alleviates aging-related phenotypes in old recipient mice
Source: Cell Res. 2025 Jan 2;35(1):45–58. doi: 10.1038/s41422-024-01057-5 (PMC11701126; doi:10.1038/s41422-024-01057-5)
Supplement: Supplementary file 5 — Supplementary Figure 5 [file 41422_2024_1057_MOESM5_ESM.pdf]

Supplementary information, Fig. S5

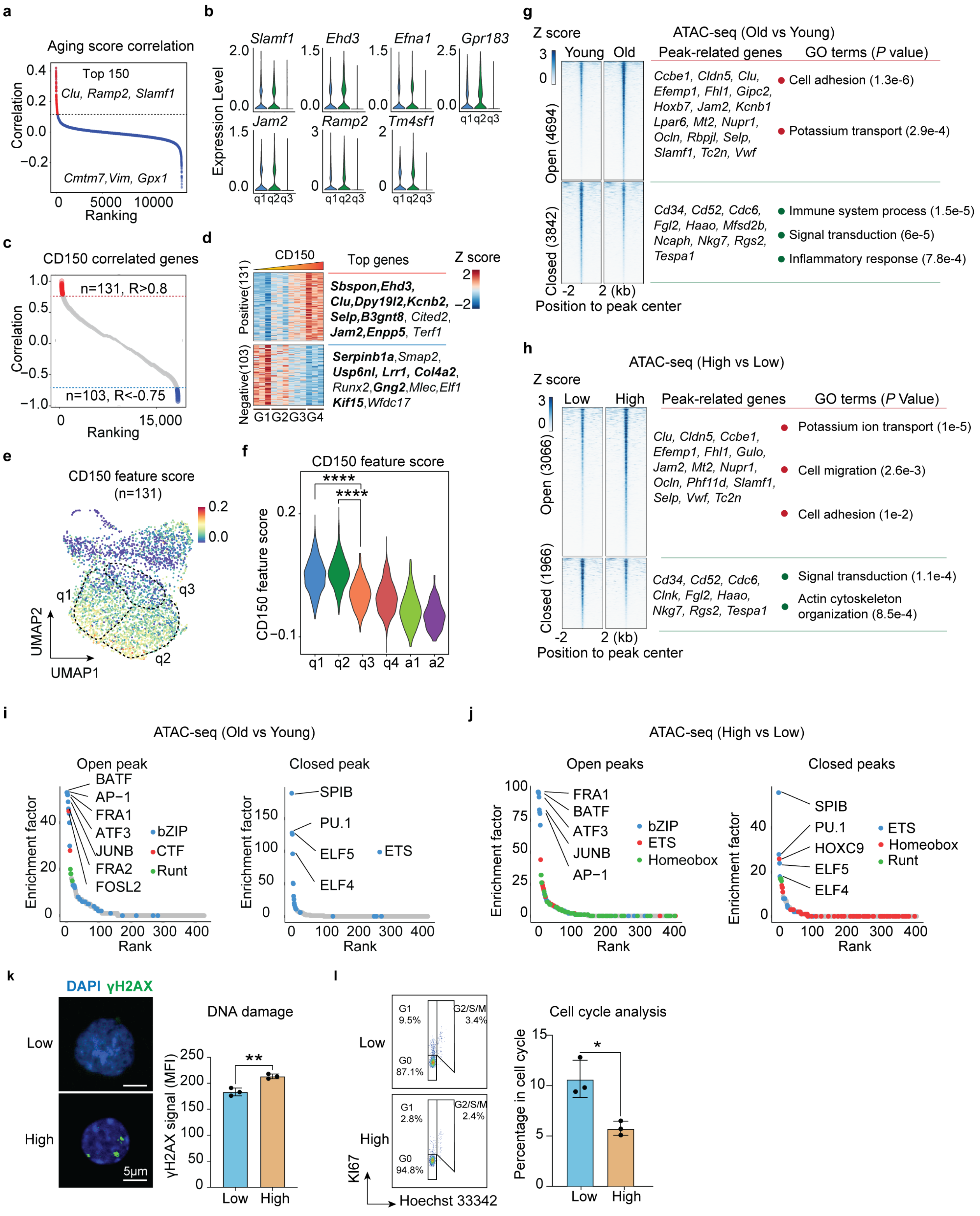

Fig. S5 CD150 can serve as an aging heterogeneity marker of old HSCs (related to Fig. 3). a Dot plot showing ranking of genes based on the correlation of their expression and aging score in scRNA-seq. The top 150 highly ranked genes were highlighted. **b** Violin plot showing expression level of potential marker genes in cluster q1, q2 and q3 by scRNA-seq. **c** Dot plot showing the ranking of genes based on their correlation with CD150 levels in expression of the four cell groups in Figure 3C measured by bulk RNA-seq. A total of 131 positively correlated genes ($R > 0.8$) and 103 negatively correlated genes ($R < -0.75$) were identified as CD150 related genes. **d** Heatmap showing expression changes of CD150-related genes with ascending CD150 level based on bulk RNA-seq. For positive correlated genes, previously identified HSC aging marker genes are highlighted. For negative correlated genes, genes that are highly expressed in young HSCs are highlighted. **e-f** Feature plot (**e**) and violin plot (**f**) showing CD150 signature scores of the six cell clusters in scRNA-seq. **g** Heatmap showing aging-related open and closed peaks, as well as representative genes near these peaks (within 5 kb of the TSS) and the corresponding enriched GO terms followed by *P* value. **h** Heatmap showing the differential ATAC-seq peaks between old $CD150^{low}$ HSCs and $CD150^{high}$ HSC, as well as representative genes near these peaks (within 5 kb of the TSS) and the corresponding enriched GO terms, followed by P value. **i-j** Dot plot showing enriched TFs in aging-related open (left) and closed (right) peaks (**i**), and differential peaks between old $CD150^{low}$ and $CD150^{high}$ HSCs (**j**). The color of dot indicates different TF families. **k** Representative images and bar plot showing the level of γH2AX in old $CD150^{low}$ and $CD150^{high}$ HSCs, $n = 3$. **l** Left, representative FACS plot showing the percentage of old $CD150^{low}$ and $CD150^{high}$ HSCs in different cell cycle phases. Right, bar graph showing the percentage of old $CD150^{low}$ and $CD150^{high}$ HSCs in active cell cycle (G1 and G2/S/M), $n = 3$. For (g) and (h), Mean ± SD, student t test, *$P<0.05$, ** $P<0.01$. For (f), Two-sided unpaired Wilcoxon test, **** $P<0.0001$.
