# Supplementary material for: Reducing functionally defective old HSCs alleviates aging-related phenotypes in old recipient mice
Source: Cell Res. 2025 Jan 2;35(1):45–58. doi: 10.1038/s41422-024-01057-5 (PMC11701126; doi:10.1038/s41422-024-01057-5)
Supplement: Supplementary file 6 — Supplementary Figure 6 [file 41422_2024_1057_MOESM6_ESM.pdf]

Supplementary information, Fig. S6

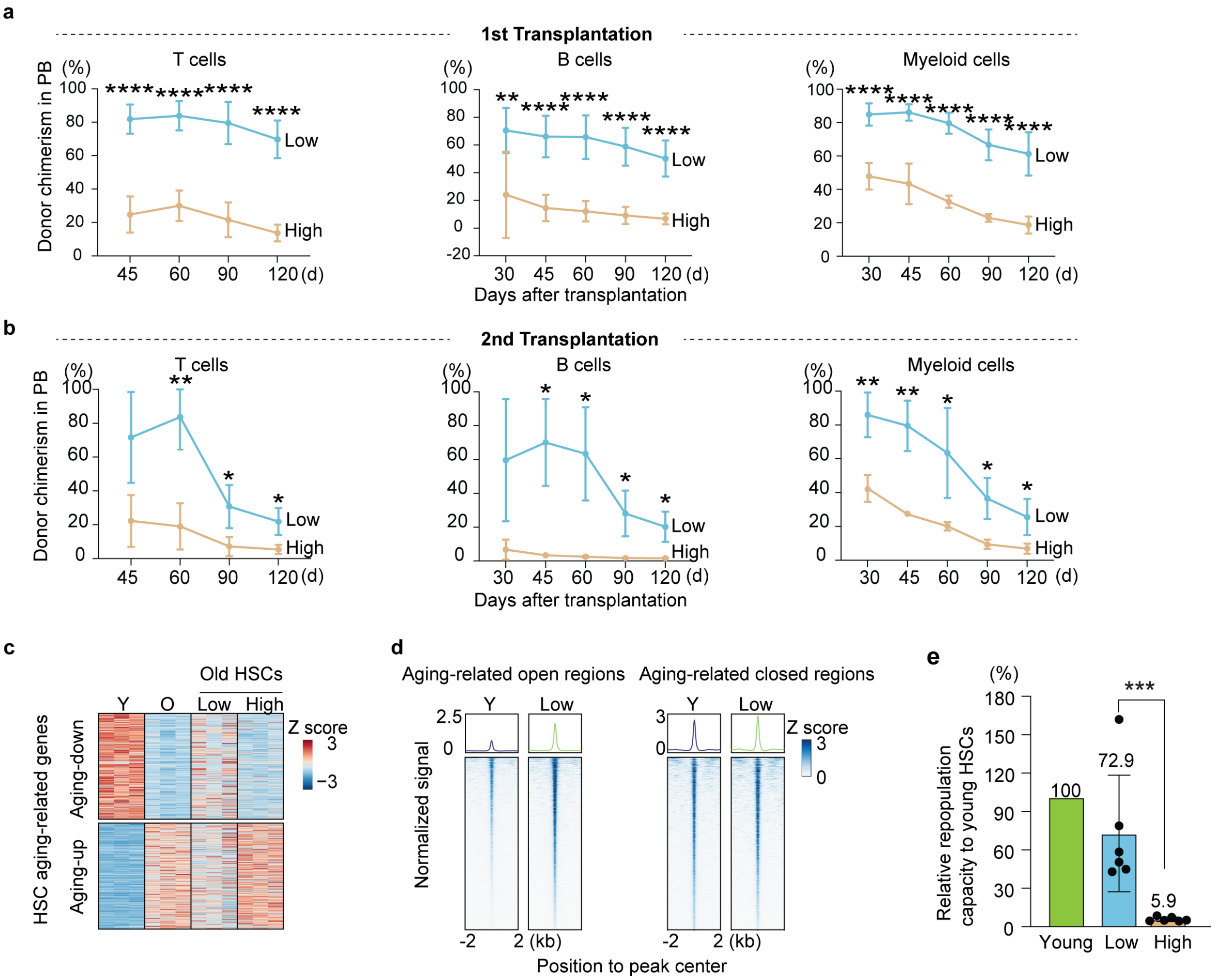

Fig. S6 Comparison of old CD150^{low}, CD150^{high}, and young HSCs at the molecular and functional levels (related to Fig. 3). a-b The peripheral blood chimerism of donor HSCs at different time after transplantation in the first (**a**) and second (**b**) competitive transplantation. B, T, and myeloid cells were analyzed individually. Mean ± SD, student t test, n = 6 for the first and n = 3 for the second transplantation. **c** Heatmap showing the expression levels of aging-related up- and down-regulated genes in young and old HSCs, as well as old CD150^{low} and CD150^{high} HSCs. **d** Heatmap and bar plot illustrating ATAC-seq signals in aging-related open and closed chromatin peaks. **e** Bar plot showing the relative repopulation capacity of old CD150^{low} HSCs and CD150^{high} HSCs to young HSCs. For each sample, their relative functionality compared to young HSCs is calculated based on their contribution to PB, normalized by the number of transplanted cells. n = 6, Mean ± SD, student t test. *P<0.05, ** P<0.01, *** P<0.001, **** P<0.0001.
