# Supplementary material for: Reducing functionally defective old HSCs alleviates aging-related phenotypes in old recipient mice
Source: Cell Res. 2025 Jan 2;35(1):45–58. doi: 10.1038/s41422-024-01057-5 (PMC11701126; doi:10.1038/s41422-024-01057-5)
Supplement: Supplementary file 7 — Supplementary Figure 7 [file 41422_2024_1057_MOESM7_ESM.pdf]

Supplementary information, Fig. S7

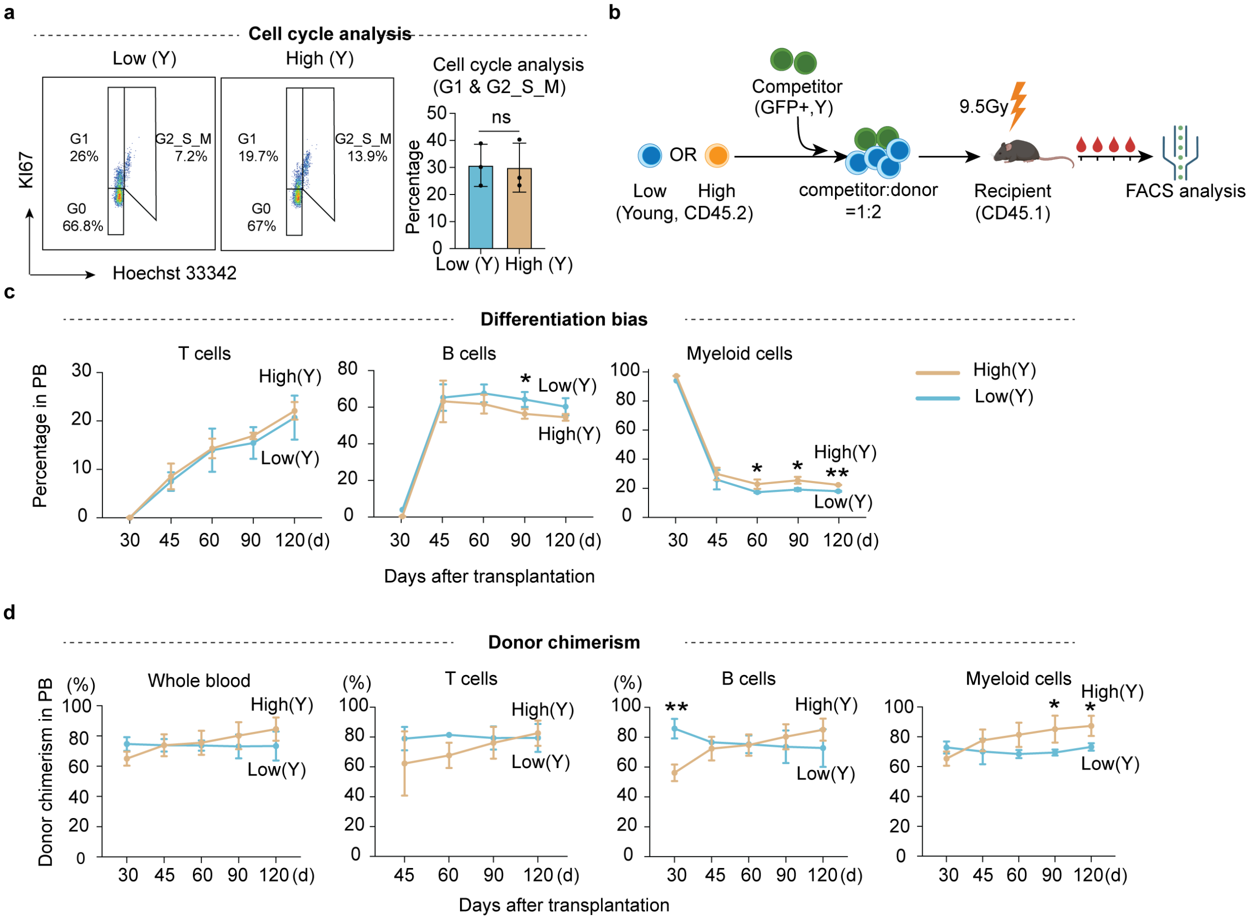

Fig. S7 Comparable function of CD150^{low} and CD150^{high} HSCs from young mice (related to Fig. 3). a Left, representative FACS plot showing the percentage of young CD150^{low} (25% lowest) and CD150^{high} (25% highest) HSCs in different cell cycle phases. Right, bar graph showing the average percentage of young CD150^{low} and CD150^{high} HSCs in active cell cycle (G1 and G2/S/M), *n* = 3. **b** Diagram illustrating the competitive transplantation for evaluating the repopulating capacity of CD150^{low} and CD150^{high} HSCs from young mice. The ratio of competitor to donor HSCs to was 1:2 (300 competitor HSCs with 600 donor HSCs). **c-d** The differentiation bias (**c**) and peripheral blood chimerism (**d**) of donor HSCs at different time after transplantation. Whole blood, T, B, and myeloid cells were analyzed. *n* = 3, Mean ± SD, student t test, **P*<0.05, ***P*<0.01, ns, not significant. The graphic of the mouse in **b** was created with BioRender.
