# Supplementary material for: Reducing functionally defective old HSCs alleviates aging-related phenotypes in old recipient mice
Source: Cell Res. 2025 Jan 2;35(1):45–58. doi: 10.1038/s41422-024-01057-5 (PMC11701126; doi:10.1038/s41422-024-01057-5)
Supplement: Supplementary file 8 — Supplementary Figure 8 [file 41422_2024_1057_MOESM8_ESM.pdf]

Supplementary information, Fig. S8

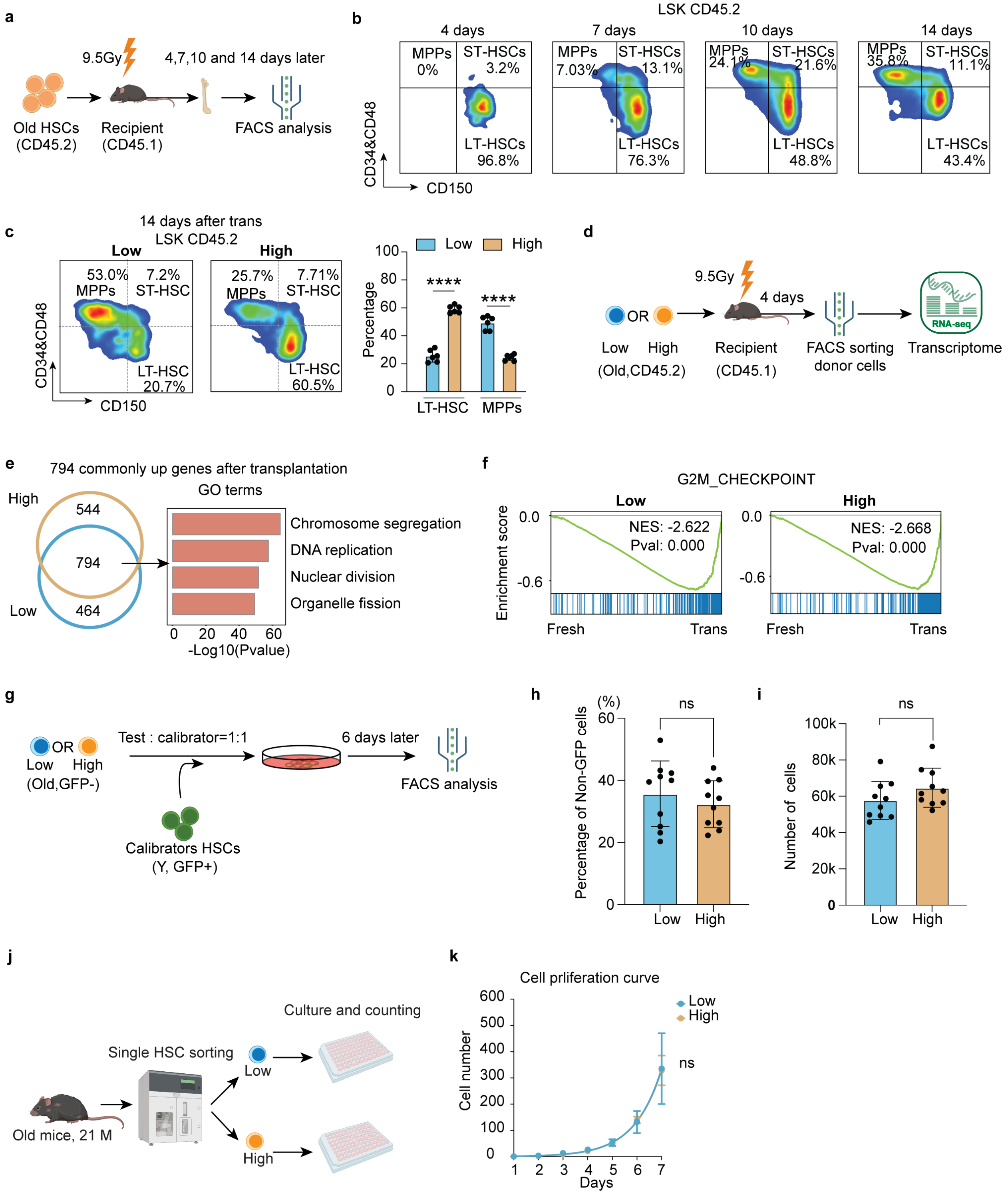

Fig. S8 Differentiation, but not self-renewal, is a major defect of old CD150^{high} HSCs (related to Fig. 4). a Diagram of individual transplantation of old HSCs to study the differentiation trajectory after transplantation. The donor HSCs derived HSPCs in bone marrow were analyzed on days 4, 7, 10 and 14 after transplantation. **b** FACS analysis showing the differentiation trajectory of old HSCs after transplantation. On the 4th day, most donor HSCs are still maintained as LT-HSCs. From 7th days the LT-HSCs started to differentiate toward MPPs. **c** Representative FACS analysis of donor HSCs derived HSPCs (left) and quantification of different cell populations after transplantation (right) on day 14, n = 3. Mean ± SD, student t test. **d** Diagram of transplantation to evaluate the difference in activation between old CD150^{low} and CD150^{high} HSCs on day 4 after transplantation. The sorted donor HSCs were collected for transcriptome analysis, n = 3. **e** Venn diagram showing the genes that were commonly activated in old CD150^{low} and CD150^{high} HSCs compared to freshly isolated old CD150^{low} and CD150^{high} HSCs 4 days after transplantation (left). Bar graph showing enriched GO terms of the 794 commonly activated genes (right). **f** GSEA analysis showing that transplanted HSCs highly express cell cycle related genes in both CD150^{low} (left) and CD150^{high} (right) HSCs when compared with freshly isolated HSCs. **g** Diagram showing the experimental design for examining HSCs proliferation *in vitro*. In each well, 50 GFP+ cell and 50 old CD150^{low} or CD150^{high} HSCs were co-cultured. The percentage and absolute number of old CD150^{low} and CD150^{high} HSCs were quantified 6 days after culture. **h** Bar graph showing comparable proliferation rate of old CD150^{low} and CD150^{high} HSCs 6 days after culture. The percentage of non-GFP HSCs was shown, n = 10. Mean ± SD, student t test. **i** Bar graph showing the absolute number of old CD150^{low} and CD150^{high} HSCs 6 days after culture, n = 10. Mean ± SD, student t test. **j** Diagram showing the workflow for evaluating the proliferation capacity of old CD150^{low} and CD150^{high} HSCs. **k** Cell proliferation curve showing the cell number change with time (1-7 days), n = 8, student t test, Mean ± SEM. **P<0.01, *** P<0.001, ns, not significant. The graphic of the mouse and equipment in **a**, **d** and **j** were created with BioRender.
