# Supplementary material for: Reducing functionally defective old HSCs alleviates aging-related phenotypes in old recipient mice
Source: Cell Res. 2025 Jan 2;35(1):45–58. doi: 10.1038/s41422-024-01057-5 (PMC11701126; doi:10.1038/s41422-024-01057-5)
Supplement: Supplementary file 9 — Supplementary Figure 9 [file 41422_2024_1057_MOESM9_ESM.pdf]

Supplementary information, Fig. S9

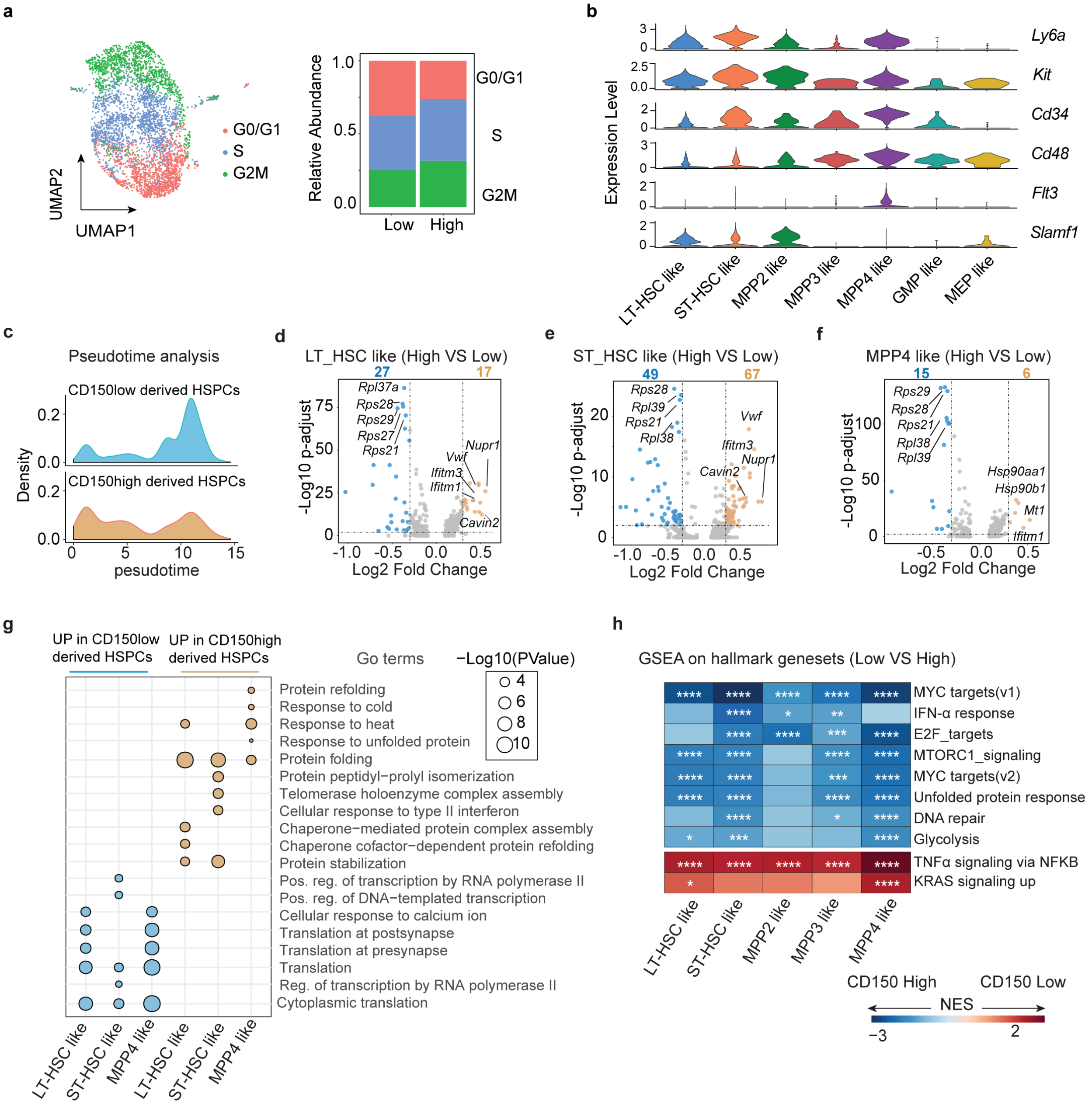

Fig. S9 Old CD150^{high} HSCs are defective in the LT-HSCs to ST-HSCs transition (related to Fig. 4). **a** UMAP and bar graph showing the distribution and percentages of cells in different cell cycle phases of HSPCs from CD150^{low} and CD150^{high} HSCs 14 days after transplantation. **b** Violin plot showing the expression pattern of cell type marker genes, including *Ly6a*, *Kit*, *Cd34*, *Cd48*, *Flt3* and *Slamf1* (CD150). **c** Density plot showing the relative abundance of cells at different pseudo time stage. **d-f** Volcano plot showing the differentially expressed genes between old CD150^{low} and

CD150^{high} HSCs derived HSPCs, including LT-HSCs-like (**d**), ST-HSCs-like (**e**) and MPP4-like (**f**) cells. **g** Dot plot showing the enriched GO terms in differentially expressed genes between old CD150^{low} and CD150^{high} HSCs derived HSPCs. The size of dot indicates the significance of enrichment. **h** Heatmap displaying the GSEA comparison between transplanted old CD150^{low} and CD150^{high} HSC-derived HPSC cell types, with red indicating activated pathways in CD150^{low} HSCs derived cell types and blue indicating activated pathways in old CD150^{high} HSCs derived cell types. *P<0.05, ** P<0.01, *** P<0.001, **** P<0.0001.
