# Supplementary material for: Reducing functionally defective old HSCs alleviates aging-related phenotypes in old recipient mice
Source: Cell Res. 2025 Jan 2;35(1):45–58. doi: 10.1038/s41422-024-01057-5 (PMC11701126; doi:10.1038/s41422-024-01057-5)
Supplement: Supplementary file 10 — Supplementary Figure 10 [file 41422_2024_1057_MOESM10_ESM.pdf]

Supplementary information, Fig. S10

a

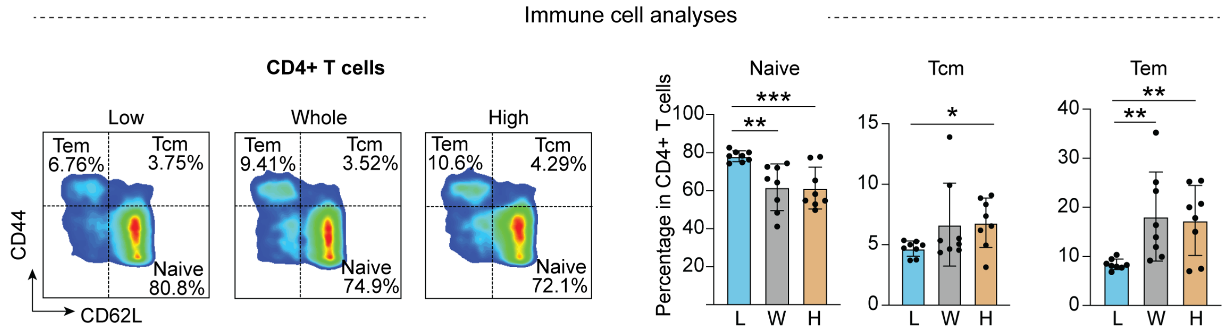

b

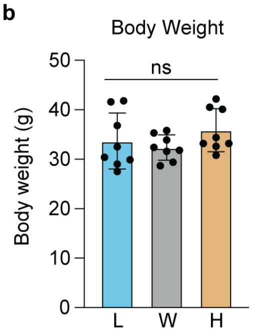

Fig. S10 Transplantation of "younger" subset old HSCs attenuates aging phenotypes of old mice (related to Fig. 5). a Representative FACS (left) analysis of naïve T cells, Tcm and Tem ratio in CD4 positive T cells of mice from different groups and their quantification (right, bar graphs). **b** Bar graph showing body weight of recipient mice from different groups. Mean ± SD, one-way ANOVA, *n* = 8, **P*<0.05, ** *P*<0.01, *** *P*<0.001, ns, not significant.
