# Supplementary material for: Reducing functionally defective old HSCs alleviates aging-related phenotypes in old recipient mice
Source: Cell Res. 2025 Jan 2;35(1):45–58. doi: 10.1038/s41422-024-01057-5 (PMC11701126; doi:10.1038/s41422-024-01057-5)
Supplement: Supplementary file 11 — Supplementary Figure 11 [file 41422_2024_1057_MOESM11_ESM.pdf]

Supplementary information, Fig. S11

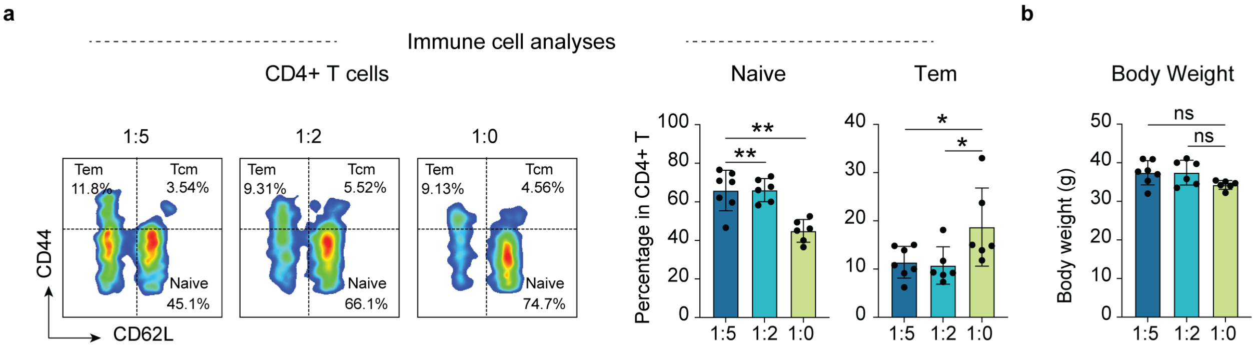

Fig. S11 Reducing dysfunctional HSCs ameliorates aging phenotypes in old mice post transplantation (related to Fig. 6). a Representative FACS (left) analysis of naïve T cells and Tem ratio in CD4 positive T cells of mice from the different groups and their quantification (right, bar graphs). **b** Bar graph showing body weight of recipient mice from different groups. n=6 for 1:5 and 1:2 group, *n* = 7 for 1:0 group; Mean ± SD, one-way ANOVA, **P*<0.05, ** *P*<0.01, ns, not significant.
